# Chronic Follicular Pharyngitis

**Published:** 1876-03

**Authors:** Bernard Tauber

**Affiliations:** Cincinnati, Ohio


### Selections.

---

### CHRONIC FOLLICULAR PHARYNGITIS

By BERNARD TAUBER, M. D., Cincinnati, O.

To the physician is rarely afforded an opportunity to see this disease until it has existed for several months or years. The patient will complain of dryness in his throat, with or without a disposition to cough or expectoration, or to clear his throat from a foreign body, feeling a lump, a hair, or a pin, etc.; some degree of hoarseness; more or less impairment of hearing and trouble in swallowing. With all these symptoms the patient will enjoy tolerable good health. At a later period all the symptoms will have incased in severity.

The causes of this affection are generally those which bring on a catarrh of the mucous membrane. Public speakers, clergymen, singers, smokers and spirit drinkers are especially liable. In medical literature this affection, from its frequency among the clergy, is known as the "clergyman's sore throat." It is not confined to this class alone, and the prevalence among them is due to exposure to draughts from open windows, and inequalities of temperature under which they often preach, etc. The appearances present themselves in small circular or irregular projections, either isolated or in clusters, varying from that of a pin-head to a small pea. Their color is deeper red than the surrounding mucous tissue These prominences consist of enlarged or hypertrophied glands. Usually we notice a narrow line of redness about the base of them. Frequently the patches are close to each other, bordered by these red lines. Sometime the mucous membrane appears sunken, and has a granulated appearance, and may occupy the edges of the arches of the palate. At this stage the patient is not annoyed much—the voice is not much affected; will be no cough, and the expectoration will be a viscid mucous. At a later stage the follicles become more enlarged, the mucous more viscid and adherent. The disease advancing to the posterior wall behind the soft palate, invading the glandula tissue of the vault of the pharynx, often strings of mucous will hang from the posterior wall of the soft palate, and extend up to the posterior nares. The patches of enlarged follicles becoming larger, their surface is often velvety. In the interspaces we may notice certain spots of superficial ulcerations, indicating a destruction of the epithelial layer of the mucous membrane. The tonsils and uvula are apt to become irregularly enlarged, and covered with a grayish or whitish secretion. The voice is often affected (though the larynx may not be implicated at first, but eventually it becomes involved), is husky at times and hoarse, or the patient may be aphonic in the morning, but when engaged in conversation the voice gradually becomes clearer. In swallowing solid food there is pain. The patient may complain also of impairment of hearing, because the lower portion of the mucous membrane of the eustachain tube is continuous with the mucous membrane of the pharynx, and the disease may thus be proapgated along the tube, and thus affect the structure of the middle ear. By a chronic thickening of these parts, the free opening of the eustachian tube is narrowed, and the access of air into the interior of the middle ear is excluded. Inflammation and enlargement of the uvula frequently coexists with chronic follicular, pharyngitis, and gives rise to a harassing cough and expectoration.

The treatment of this affection must be a chronic one, and seldom the patient will submit to it; therefore it is not always so successful as one would expect. These cases require constitutional and local remedies, and especially the latter. Sometimes the effects are very prompt, again very slow. In using astringent solutions the pharynx should be washed out by a syringe or a spray, to detach the strings of mucous adherent to the mucous membrane. This is of great importance. It is good policy not to commence with a strong solution; and apply with a small camel hair pencil, or a small piece of cotton held in a pair of pharyngeal forceps, and use a solution of nitrate of silver 30 grs. to the ounce, and increasing it to 60, 120, and 240 grs. to the ounce. The solid stick is employed when we desire to produce a destruction of the parts and maintain it in contact for some seconds.

At the clinics and hospitals in Europe, nitrate of silver is always used in diseases of the pharynx. Should we fail with this treatment, we may adopt the plan to split each follicle with a point of the knife, touching it with a crystal of nitrate of siver. We employ in addition to it the spray of solutions of tannic acid, alum, sul. zinc, or sulp. copper, etc. In order to keep up the astringent effect, we can also assist the local treatment by the use of counter-irritations. Some authorities recommend, in obstinate cases, in addition to the constitutional treatment, the use of the iodide of potash ; sometimes the bichloride of mercury in small doses is administered with the iodide of potash, even if there is no syphilitic taint to expect.—*Cincinnati Medical News*.

